# Prolonged Corrected QT Interval Is Associated with Lower Incidence of Maternal Hypotension During Spinal Anesthesia in Cesarean Delivery: A Prospective Observational Study

**DOI:** 10.3390/medicina61111925

**Published:** 2025-10-27

**Authors:** Hee-Sun Park, Dong-Min Jang, Jong Yeon Park, Won Uk Koh, Woo-Jong Choi

**Affiliations:** Department of Anesthesiology and Pain Medicine, Asan Medical Center, University of Ulsan College of Medicine, Seoul 05505, Republic of Korea; heespark@amc.seoul.kr (H.-S.P.);

**Keywords:** anesthesia, spinal, cesarean section, electrocardiography, hypotension, long QT syndrome

## Abstract

*Background and Objectives*: Spinal anesthesia is a common anesthetic method for cesarean delivery. However, it is associated with spinal hypotension, which can negatively impact both the mother and the fetus. We hypothesized that parturients with preoperatively prolonged corrected QT interval (QTc) would have a lower incidence of developing spinal hypotension. *Materials and Methods*: This prospective observational study analyzed eighty-five parturients undergoing cesarean delivery. The participants were divided into two groups based on their baseline QTc, which was measured automatically using a patient monitor in the operating room rather than using a standardized 12-lead electrocardiogram: <440 ms (*n* = 42) or ≥440 ms (*n* = 43). Following combined spinal-epidural anesthesia, the incidence of spinal hypotension until delivery was analyzed and the vasopressor requirements within 30 min were compared between the QTc groups. The area under the receiver operating characteristic curve was measured to identify the optimal QTc cut-off for predicting spinal hypotension. *Results*: Spinal hypotension was observed in 37/43 parturients (86.0%) with QTc < 440 ms, compared to 17/42 (40.5%) with QTc ≥ 440 ms (*p* < 0.001). The total amount of phenylephrine significantly differed between groups (300 μg [100–400] vs. 100 μg [0–300], *p* = 0.009). The area under the ROC curve for spinal hypotension prediction was 0.75 (95% confidence interval [CI] 0.64–0.86). The optimal QTc cut-off interval, determined using the maximum Youden index (J = 0.510), which corresponded to the best combination of sensitivity and specificity, was 441 ms. *Conclusions*: These preliminary patient-monitor-based findings indicate an association between preoperative QTc and spinal hypotension, which should be validated using standardized electrocardiographic methods.

## 1. Introduction

Cesarean delivery is commonly performed under spinal anesthesia because it provides several advantages over general anesthesia. Spinal anesthesia allows mothers to remain awake during delivery and avoids complications associated with general anesthesia, such as failed endotracheal intubation or aspiration pneumonia [1,2]. In addition, it offers rapid onset, a denser surgical block, and effective postoperative analgesia. However, spinal hypotension is a frequent complication of spinal anesthesia, with a reported incidence as high as 71% [3]. Spinal hypotension may cause maternal nausea, vomiting, and dizziness, and may reduce placental blood flow, leading to fetal acidosis [4]. No factor definitively predicts spinal hypotension, although various indicators have been assessed. Additionally, most indicators are too complex or costly for routine use [5,6,7].

The QT interval reflects ventricular repolarization and is modulated by autonomic nervous system activity [8]. The corrected QTI (QTc) of patients with pre-eclampsia—a condition associated with sympathetic hyperactivity—is longer than that of normotensive parturients [9,10]. These electrocardiographic alterations may result from increased sympathetic drive, hypertension, and hypocalcemia [11,12,13]. In this context, parturients with preeclampsia who had a prolonged QTc tended to experience less spinal hypotension than normotensive women with shorter baseline QTc values [10]. These findings suggest that prolonged QTc may reflect increased sympathetic activity and autonomic dysregulation, thereby paradoxically leading to relative resistance to sympathetic blockade induced by spinal anesthesia.

Based on these observations, we formulated an exploratory hypothesis that normotensive parturients with longer baseline QTc values may exhibit a lower incidence of spinal hypotension. The value of 440 ms used as the cut-off values, which represents the traditional threshold for diagnosing long QT syndrome [14,15]. Therefore, we investigated the association between preoperative QTc and the incidence of hypotension during spinal anesthesia for cesarean delivery. The secondary outcome was to prospectively compare vasopressor requirements according to preoperative QTc values.

## 2. Materials and Methods

This prospective observational study was conducted in a single, tertiary medical center in Seoul, Republic of Korea. The study design was approved by the Institutional Review Board (IRB) of Asan Medical Center (protocol number 2018-0962) prior to study initiation. The IRB waived the requirement for written informed consent due to the nature of the study. This study was conducted in accordance with the Declaration of Helsinki. It was registered with the Clinical Research Information Service of South Korea (KCT0009375).

### 2.1. Patients and Eligibility Criteria

Pregnant women scheduled for elective cesarean delivery between October 2018 and September 2019 were enrolled in this study. Patients with an American Society of Anesthesiologists physical status of 2 and aged >20 and ≤40 years old were included. The exclusion criteria were cardiac diseases (valvular heart disease, conduction abnormalities, or cardiovascular disease that can affect the blood pressure of parturients), hypertensive disorders complicated pregnancy (gestational hypertension, preeclampsia, eclampsia, superimposed preeclampsia on chronic hypertension, chronic hypertension), diabetes mellitus, placenta previa, and multiple pregnancy. We also excluded patients with incomplete electrocardiography (ECG) data obtained through monitoring in 1-min intervals in the operating room and those with incomplete electrical medical records that did not accurately describe patient information such as comorbidities or medication use.

### 2.2. Data Sources

All parturients received routine monitoring, including 3-lead ECG, non-invasive blood pressure (NIBP), and pulse oximetry. The baseline parameters were recorded in the supine position before anesthetic induction. ECG, oxygen saturation (SpO_2_), systolic blood pressure (SBP), diastolic blood pressure (DBP), mean blood pressure (MBP), and heart rate (HR) were monitored by one anesthesiologist. Through lead II, continuous ECG waveforms were observed, with the values obtained from ECG analysis before anesthesia set as the baseline values. NIBP was measured in 1-min intervals to treat spinal hypotension as the established protocol for 30 min. Patient data were collected in the supine position in 1-min intervals after combined spinal–epidural anesthesia (CSEA). The QT interval, QTc, and the change from baseline (Δ) in QTc were measured automatically as 1-min average values from the patient monitor (IntelliVue MP70, Philips Medizin Systeme Böblingen GmbH, Böblingen, Germany) connected to the parturient with ECG lead. The QT interval measured from the patient monitor was corrected for heart rate using Bazett’s formula (QTc = QT interval/(RR interval)^1/2^). According to the baseline default QTc value, the parturients were divided into two groups: QTc ≥ 440 ms vs. QTc < 440 ms. This study defined baseline QTc ≥ 440 ms as prolonged based on the reference values in several studies [14,15,16].

### 2.3. CSEA and Management in the Operating Room

CSEA was performed according to the following protocol. In all cases, CSEA was performed at the L3–4 or L4–5 vertebral interspaces with the patient in the left lateral decubitus position. After skin infiltration with lidocaine, an 18-gauge Tuohy needle was placed in the epidural space using the loss of resistance technique. Once a free flow of clear cerebrospinal fluid was observed in the 27-gauge Sprotte spinal needle inserted through the Tuohy needle using the needle-through-needle technique, hyperbaric 0.5% bupivacaine (8 mg) with fentanyl (15 μg) was administered intrathecally. After placement and fixation of an epidural catheter, patients were immediately turned supine from the left lateral decubitus position. Administration of local anesthetic via the epidural catheter was performed only for postoperative analgesia, and epidural administration of local anesthetics including the test dose was not performed during the study period. Oxygen was administered via a facemask at 5 L/min. The upper sensory level of the block to cold was measured using alcohol swabs 10 min after spinal injection. To prevent maternal spinal hypotension, crystalloid was infused rapidly (16–20 mL/kg/h) and the uterus was displaced to the left. Spinal hypotension was defined as a decrease in SBP to <80% of baseline value and was treated with intravenous phenylephrine 100 μg as required. BP was assessed in 1-min intervals. If hypotension did not respond to phenylephrine, ephedrine 5 mg was administered intravenously. The primary endpoint was the time of delivery. The incidence of spinal hypotension was evaluated as the primary outcome, occurring after the administration of the spinal anesthetic until delivery. After delivery, intravenous carbetocin 100 μg was administered slowly over 1 min. This study was completed 30 min after the spinal injection, at which point data collection ended, and routine care was continued.

### 2.4. Sample Size

The sample size was based on a preliminary examination of 47 clinical cases in our department for which we used the same anesthetic management protocol. In the preliminary study, we observed that hypotension during spinal anesthesia occurred in 16 of 21 (76.2%) women with QTc < 440 ms and in 12 of 26 (46.2%) women with QTc ≥ 440 ms (*p* = 0.037). Using the software package PASS 15.0 (NCSS, LLC., Kaysville, UT, USA), sample sizes of 40 in each group were deemed to achieve 80.743% power to detect a difference between a group proportion of 0.30. A two-sided Z-test with unpooled variance was used as the test statistic, and the significance level was set at 0.05. We included 43 patients per group to account for a possible dropout rate of 5%.

### 2.5. Statistical Analysis

Data are expressed as means (standard deviation [SD]) or medians (Q1–Q3) for continuous variables and as numbers (percentages) for categorical variables. We compared the patient characteristics and baseline hemodynamic data between groups using independent *t*-tests or Mann–Whitney U tests for continuous variables and chi-squared or Fisher’s exact tests for categorical variables.

We evaluated the validity of the baseline QTc cut-off for predicting spinal hypotension by analyzing the operating characteristic (ROC) curve. We also calculated the sensitivity, specificity, positive predictive value (PPV), and negative predictive value (NPV) of the baseline QTc for the incidence of hypotension. The area under the ROC curve (AUC) was used for assessing overall predictive performance of the QTc cut-off. The optimal cut-off threshold was determined by maximizing Youden’s index (J = sensitivity + specificity − 1). The corresponding J value, sensitivity, and specificity are reported. No internal validation such as bootstrapping or cross-validation was performed.

External validation was performed for statistical verification of the predictive performance according to the baseline QTc cut-off. For this purpose, we collected data from 80 parturients who met the study inclusion criteria at our department for one month in May 2022. A total of 44 of these 80 parturients had baseline QTc of <440 ms and the rest had baseline QTc of ≥440 ms. The demographic data of the external validation set are provided as supplementary data (Supplementary Table S1).

We used univariate and multivariate logistic regression analysis to identify the risk factors associated with spinal hypotension. All variables with *p* < 0.1 in the univariate analysis were included in the multivariate analysis using the stepwise variable selection method. All statistical analyses were performed using SAS version 9.4 (Cary, NC, USA). *p* < 0.05 was considered statistically significant.

## 3. Results

**Figure 1:**
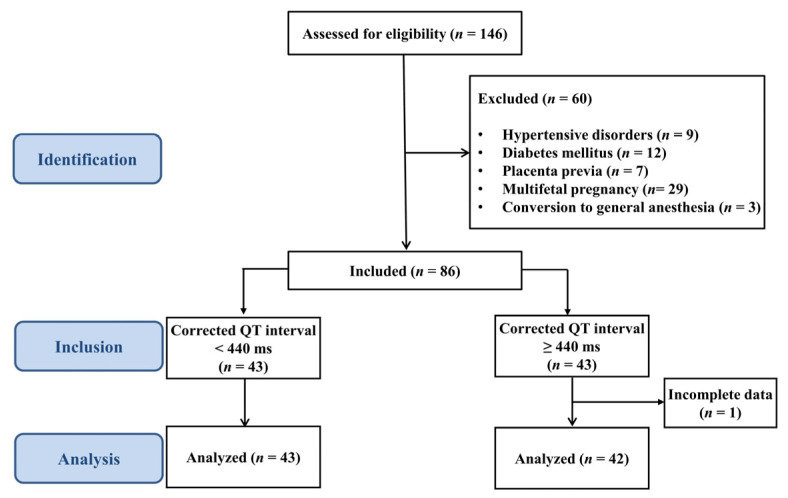
Flow diagram.

A total of 146 parturients undergoing elective cesarean delivery under CSEA were assessed for eligibility. A total of 60 of these 146 parturients were excluded (Figure 1). One parturient with incomplete ECG data during cesarean delivery was also excluded from the analysis. Finally, 43 parturients with QTc < 440 ms and 42 parturients with QTc ≥ 440 ms were included in the analysis (Figure 1). Table 1 presents the maternal and neonatal demographic data, preoperative electrolyte levels, and anesthetic and surgical data. There were no significant differences between the two groups in terms of baseline characteristics.

### 3.1. Comparisons of Hemodynamic Parameters Between Groups

The baseline hemodynamic parameters did not significantly differ between the two groups (Table 2). Spinal hypotension until the time of delivery was observed in 37 of 43 (86.0%) women with QTc < 440 ms and in 17 of 42 (40.5%) women with QTc ≥ 440 ms (*p* < 0.001). The incidence of hypotension at 30 min after CSEA was also significantly higher in the QTc < 440 ms group (38 of 43, 88.4%) than in the QTc ≥ 440 ms group (28 of 42, 66.7%; *p* = 0.016).

### 3.2. Predictive Performance According to the Baseline QTc

**Figure 2:**
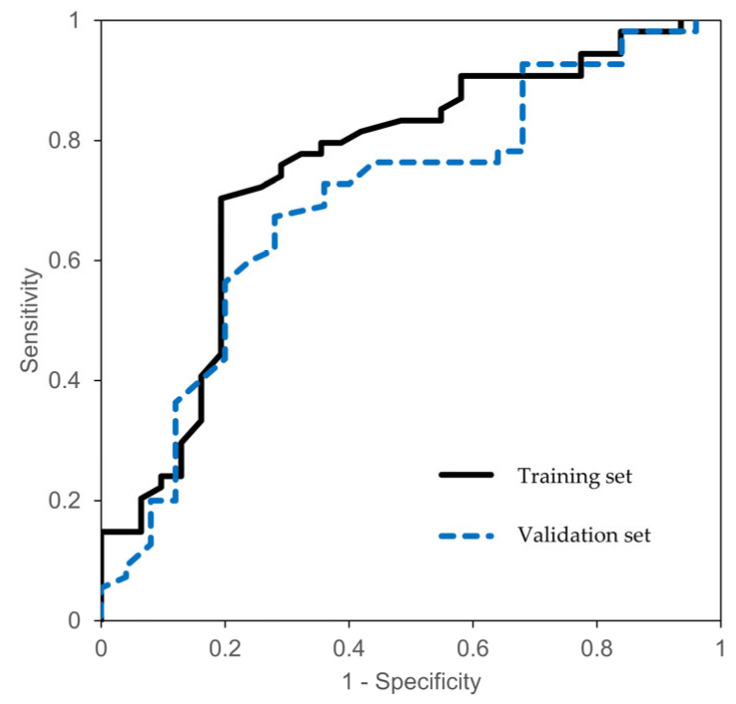
Receiver operating characteristic (ROC) curves for predicting spinal hypotension based on the baseline corrected QT interval cut-off. The areas under the ROC curves for the training set (black line) and the external validation set (blue dashed line) were 0.75 (95% confidence interval [CI] 0.64–0.86) and 0.70 (9% CI 0.57–0.83), respectively. The optimal cut-off value in the training set determined by the Youden index was 441 ms which yielded a sensitivity of 70.3% and a specificity of 80.6%.

Table 3 summarizes the predictive performance for various baseline QTc cut-off values for spinal hypotension. The analyses were performed using threshold of 440 ms, 450 ms, and 460 ms. The sensitivity in predicting spinal hypotension within 15 min after anesthesia was significant at all three cut-off values (*p* < 0.05). However, the specificity in identifying spinal hypotension with 15 min was the most significant at the 440 ms cut-off. In the external validation set, the specificity at the 440 ms cut-off remained significant, whereas it was not significant at the 450 ms threshold. The area under the ROC curve of the training set was 0.75 (95% CI, 0.64–0.86) when using the baseline QTc cut-off as a predictor of maternal hypotension (Figure 2). According to the Youden index analysis, the optimal cut-off value was 441 ms, which yielded the highest Youden index (J = 0.510), providing the best trade-off between sensitivity and specificity for predicting hypotension. Table 4 presents the results of multivariate analyses of the risk factors for spinal hypotension. Regardless of adjustment for covariates, the baseline QTc was significantly related to the incidence of hypotension.

### 3.3. Vasopressor Requirement

The total dose of phenylephrine administered within 30 min for spinal hypotension treatment was significantly higher in the baseline QTc < 440 ms group than that in the baseline QTc ≥ 440 ms group (300 μg [100–400] vs. 100 μg [0–300]; *p* = 0.009). No significant differences in the use of ephedrine were observed between the two groups. Spearman’s rank correlation analysis revealed a significant negative correlation between the baseline QTc and the total amount of phenylephrine (correlation coefficient, −0.315; *p* = 0.003).

## 4. Discussion

We found association between a prolonged preoperative QTc (≥440 ms), as automatically measured with a patient monitor, and a lower incidence of spinal hypotension in parturients compared with those without a prolonged QTc. The area under the ROC curve of the training set was 0.75 (95% CI 0.64–0.86) when baseline QTc cut-off value was used to predict maternal hypotension. The Youden index analysis showed that the optimal QTc cut-off value for predicting hypotension was 441 ms in the training set. Furthermore, the vasopressor requirement within 30 min following spinal anesthesia was significantly lower in the prolonged QTc group (*p* = 0.009).

The criteria for QT interval prolongation differ depending on sex and heart rate [8] and various reference values have been suggested in previous studies [15,17]. In this study, the value of 440 ms used as the cut-off value, which represents the traditional threshold for diagnosing long QT syndrome [14,15,16] and the reference point for “borderline QT prolongation” [18,19]. The QTc gradually increases throughout pregnancy, with an average prolongation of approximately 20 ms during the second and third trimesters compared with that of non-pregnant women [20]. Pregnancy involves major cardiovascular adjustments that affect ventricular repolarization and sympathetic activation, which may explain the QTc prolongation in the third trimester [20]. For example, eccentric cardiac hypertrophy during pregnancy, autonomic nervous system alterations, or sustained sympathetic activation during pregnancy may contribute to QTc changes [19,20,21].

Considering these physiological changes, we hypothesized that parturients with a longer QTc are relatively resistant to the sympathetic blockade induced by spinal anesthesia due to their slightly enhanced sympathetic tone. This hypothesis is further supported by the findings of a study in parturients with pre-eclampsia, in whom QTc—associated with sympathetic overactivity—was substantially prolonged but normalized after spinal anesthesia as sympathetic tone decreased [9]. Therefore, although we analyzed the optimal baseline QTc threshold, the aim of this study was not to determine the exact diagnostic cut-off value but rather to suggest the association between baseline QTc obtained from routine patient monitoring and the incidence of spinal hypotension. The use of QTc offers a simple intuitive, and cost-free parameter that may help anesthesiologists anticipate hypotension and optimize vasopressor use during cesarean delivery.

Several methodological limitations of our QTc measurement should be considered prior to drawing physiological implications from these findings. Automated QTc measurements from patient monitors were intermittently and not continuously obtained and were thus highly susceptible to artifacts such as signal noise, baseline wander, or poor lead contact. Although our results were externally validated, the use of other QTc formulas or monitors from other manufacturers may have yielded different results [21]. Therefore, our findings should be interpreted as hypothesis-generating observations rather than evidence of a specific physiological mechanism.

We divided the parturients into two groups according to the baseline QTc of 440 ms. However, it was also necessary to determine which cut-off value would provide the most appropriate discriminatory performance. The ROC curve analysis in this study showed significant results for baseline QTc cut-offs of not only 440 ms but also 450 ms and 460 ms in the assessment of the sensitivity to spinal hypotension. The relationship between baseline QTc cut-off and the incidence of spinal hypotension would have been clearer if the incidence had decreased as the baseline QTc was prolonged; however, this was not observed. Therefore, we analyzed the optimal cut-off, considering the tradeoff between sensitivity and specificity.

Postpartum hemodynamic changes could affect the development of hypotension; however, we set the time of newborn delivery as the primary end point to analyze the incidence of hypotension. The effect of carbetocin administered after delivery on QTc could not be completely excluded [22]. Nevertheless, the incidence of hypotension also differed significantly even 30 min after CSEA.

We excluded parturients with hypertensive disorders such as preeclampsia to eliminate the potential confounding factors of altered autonomic regulation. Hypertension in preeclampsia is associated with sympathetic and cholinergic imbalance, resulting in increased systemic vascular resistance and arterial blood pressure [9,11]. This abnormal sympathetic regulation may directly affect ventricular repolarization [23,24] and QTc prolongation before cesarean delivery has been reported in such patients in a meta-analysis [20]. In contrast, our study included normotensive parturients, thereby excluding these confounding hypertensive mechanisms and allowing exploration of the association between baseline QTc and spinal hypotension in physiologically normal pregnancies without gestational hypertension.

In addition to the influence of hypertensive disorders, several physiological factors during pregnancy may also affect ECG alteration. Abdominal organ enlargement, hormonal and sympathetic changes, and increased impedance of tissues located between the heart and the body surface can increase the QT interval during pregnancy [25]. These effects are likely a natural physiological process in which a pregnant woman prepares for the course of pregnancy or hemodynamic risk such as childbirth. Because the physiological QTc prolongation that normally occurs during pregnancy may be omitted in parturients with a shorter QTc (<440 ms), these patients might be more vulnerable to hypotension following sympathetic blockade induced by spinal anesthesia.

The difference in vasopressor requirement between groups may also be influenced by the pharmacologic characteristics of phenylephrine. At clinical doses, phenylephrine has a potent direct α1-effect with virtually no β-effects [26]. Compared with other vasopressors, phenylephrine has an immediate onset but a shorter duration than ephedrine and metaraminol [26], which requires multiple bolus administrations to treat spinal hypotension. When given at higher doses, it may induce baroreceptor-mediated bradycardia with a subsequent reduction in maternal cardiac output [27]. Because increased doses of phenylephrine may be associated with an increase in bradycardia, a bolus dose of 100 μg is commonly used [28]. Dependence on high doses of phenylephrine for restoring blood pressure may lead to low cardiac output if no other maneuvers are used. We used phenylephrine as a vasopressor with non-pharmacological maneuvers for hypotension prevention such as intravenous fluid administration and left lateral uterine displacement. However, when the drug is administered repeatedly based only on the occurrence of hypotension and if the hypotension is not effectively treated, the characteristics of this drug should also be considered. While the correlation coefficient (−0.315, *p* = 0.003) obtained through Spearman’s rank correlation analysis related to the vasopressor requirement was rather low, it can be meaningful in that it shows the opposite direction to the magnitude of the baseline QTc value.

This study has several limitations. First, the QT measurement and heart rate correction formulas are inherently restricted. The QTc values were automatically calculated using patient monitors with Bazett’s formula, which accurately corrects the values only within a heart rate range of approximately 60–100 beats/min—a range that is uncommon in pregnancy [29]. Using Bazett’s correction may have led to inaccurate QTc values because the maternal heart rate is typically elevated during pregnancy, and patient anxiety can further affect the QTI [30,31]. Alternative correction formulas, such as Fridericia and Framingham formulas, could be used to increase the accuracy of the rate correction and provide more reliable results in this population; however, these options were not available in the monitoring system used. Moreover, the Fridericia and Framingham corrections yield superior prognostic value for mortality than Bazett’s correction [21]. Few studies have evaluated QT correction formulas in pregnant women. As such, researchers should use 12-lead ECGs with manual QT interval verification, and cardiologists or trained personnel should compare different correction methods using dedicated analysis software in future studies. Second, the definition of spinal hypotension varies among studies [32,33] and may affect outcomes. In particular, the baseline blood pressure value used to define hypotension is critical. In this study, the first SBP measurement obtained after entering the operating room was used as the reference value. If the average value of multiple blood pressure measurements [34] or the baseline value obtained in the preoperative holding area were used, different results could have been observed. Furthermore, the results may differ depending on whether hypotension is defined using MBP rather than SBP, a reduction threshold other than 20%, or a fixed cut-off such as ≤90 mmHg. A sensitivity analysis applying these alternative definitions could potentially yield different estimates of incidence and association. However, because methods for measuring MBP were not routinely used in clinical practice until recent decades, it is unlikely that MBP-based definitions of obstetric hypotension will be adopted without considerably more supportive data [26]. Third, the analytical framework was limited. A strong crude association was observed between the baseline QTc value and the incidence of spinal hypotension (crude risk ratio 0.47); however, the observational design and sole reliance on stepwise logistic regression limit causal inference. Additional robustness assessments—such as predefined covariate adjustment, model calibration, and sensitivity analyses—were not performed. Fourth, the QTc cut-off value was determined using Youden index for exploratory predictive purpose. Modeling QTc as a continuous predictor may provide a more refined understanding of its relationship of QTc with hypotension, an aspect that should be considered in future research. Finally, internal validation procedures such as bootstrapping, cross-validation or decision-curve analysis of clinical utility were not performed. This was primarily due to the exploratory nature of this study and the limited sample size. As such, these additional analyses were statistically unstable. Nevertheless, the external validation served as a robust test of reproducibility, suggesting reasonable stability and generalizability of our findings beyond the original dataset. Despite these limitations, this study provides preliminary evidence of an association between prolonged QTc values and a lower incidence of spinal hypotension, warranting further larger, prospectively designed studies.

## 5. Conclusions

In this observational study, a longer preoperative QTc value obtained from a patient monitor was observed to be associated with a significantly lower incidence of spinal hypotension during cesarean delivery. This association suggest a physiological marker for maternal hemodynamic risk prediction during spinal anesthesia if validated in studies using standardized ECG methodologies.

## Figures and Tables

**Table 1:** Patient characteristics.

	QTc < 440 ms	QTc ≥ 440 ms	
Variables	(*n* = 43)	(*n* = 42)	*p*-Value
Age (years)	35 (3.3)	34 (3.3)	0.468
Weight (kg)	69.0 (8.5)	69.0 (7.9)	0.995
Height (cm)	161.6 (5.6)	161.4 (5.4)	0.850
Gestational age (weeks)	38.5 (0.9)	38.6 (0.7)	0.704
Diagnosis, *n* (%)			
Cephalopelvic disproportion	13 (30.2)	18 (42.9)	0.227
Previous cesarean delivery	15 (34.9)	12 (28.6)	0.532
Uterine myoma	5 (11.6)	5 (11.9)	1.000
Breech presentation	6 (14.0)	2 (4.8)	0.265
Others	8 (18.6)	8 (19.1)	0.958
Potassium (mmol/L)	4.2 (0.3)	4.2 (0.3)	0.586
Calcium (mg/dL)	9.0 (0.4)	9.1 (0.4)	0.631
Surgical time (min)	42.3 (8.3)	43.5 (7.6)	0.483
Anesthesia time (min)	61.6 (7.6)	62.2 (6.3)	0.701
CSEA to delivery interval (min)	17.0 (4.5)	16.6 (3.7)	0.615
Apgar score at 1 min	8 [8, 9]	8 [8, 9]	0.132
Apgar score at 5 min	9 [9]	9 [9–9)	0.128

Data are presented as mean (standard deviation), median [Q1–Q3], or number (percentage). QTc, corrected QT interval; ms, milliseconds; CSEA, combined spinal–epidural anesthesia.

**Table 2:** Baseline hemodynamic parameters of the patients.

	QTc < 440 ms	QTc ≥ 440 ms	
Variables	(*n* = 43)	(*n* = 42)	*p*-Value
Baseline HR (beat/min)	76.3 (11.5)	78.0 (9.6)	0.463
Baseline SBP (mmHg)	118.1 (15.3)	114.3 (11.1)	0.196
Baseline DBP (mmHg)	74.1 (13.0)	72.8 (10.3)	0.602
Baseline MBP (mmHg)	87.3 (12.8)	85.2 (10.0)	0.390
Baseline QT interval (ms)	378.6 (25.2)	401.3 (24.4)	<0.001
Sensory block level, *n* (%)			1.000
T4	41 (95.4)	41 (97.6)	
T6	2 (4.7)	1 (2.4)	

Values are expressed as mean (standard deviation) or number (percentage). QTc, corrected QT interval; ms, milliseconds; HR, heart rate; SBP, systolic blood pressure; DBP, diastolic blood pressure; MBP, mean blood pressure.

**Table 3:** Predictive performance of baseline QTc for spinal hypotension until the time of delivery after CSEA.

	440 ms Cut-Off		450 ms Cut-Off		460 ms Cut-Off	
	Estimate (95% CI)	*p*-Value	Estimate (95% CI)	*p*-Value	Estimate (95% CI)	*p*-Value
Study subjects						
Sensitivity	0.685 (0.561–0.809)	0.007	0.759 (0.645–0.873)	<0.001	0.852 (0.757–0.947)	<0.001
Specificity	0.807 (0.667–0.946)	<0.001	0.710 (0.550–0.870)	0.020	0.452 (0.276–0.627)	0.590
PPV	0.861 (0.757–0.964)		0.820 (0.714–0.927)		0.730 (0.621–0.840)	
NPV	0.595 (0.447–0.744)		0.629 (0.469–0.789)		0.636 (0.435–0.837)	
External validation						
Sensitivity	0.684 (0.564–0.805)	0.005	0.772 (0.663–0.881)	<0.001	0.930 (0.864–0.996)	<0.001
Specificity	0.720 (0.544–0.896)	0.028	0.400 (0.208–0.592)	0.317	0.280 (0.104–0.456)	0.028
PPV	0.848 (0.744–0.952)		0.746 (0.635–0.857)		0.747 (0.645–0.848)	
NPV	0.500 (0.337–0.663)		0.435 (0.232–0.637)		0.636 (0.352–0.921)	

QTc, corrected QT interval; CSEA, combined spinal–epidural anesthesia; ms, milliseconds; CI, confidence interval; PPV, positive predictive value; NPV, negative predictive value.

**Table 4:** Univariate and multivariate stepwise logistic regression analysis for spinal hypotension.

	Univariate Regression			Multivariate Stepwise Regression		
	OR	95% CI	*p*-Value	OR	95% CI	*p*-Value
Baseline QTc	0.964	0.944–0.984	<0.001	0.961	0.940–0.983	<0.001
Age	1.188	1.028–1.374	0.020	1.219	1.041–1.427	0.014
Weight	0.995	0.942–1.051	0.865			
Height	0.968	0.892–1.049	0.423			
Gestational age	0.696	0.385–1.261	0.232			
Calcium	0.479	0.095–2.411	0.372			
Potassium	0.491	0.145–1.666	0.254			
Baseline SBP	0.991	0.959–1.024	0.578			
Baseline HR	0.959	0.917–1.002	0.062			

QTc, corrected QT interval; OR, odds ratio; CI, confidence interval; SBP, systolic blood pressure; HR, heart rate. Percent increase in the incidence of hypotension is associated with a one-unit increase in baseline QTc, after controlling for confounders.

## Data Availability

The data that support the findings of this study are available from the corresponding author upon reasonable request.

## Supplementary Materials

The following supporting information can be downloaded at: https://www.mdpi.com/article/10.3390/medicina61111925/s1, Table S1: Patient characteristics of external validation set.

## Abbreviations

The following abbreviations are used in this manuscript:

CSEA	Combined spinal–epidural anesthesia
QTc	Corrected QT interval
