# Supplementary material for: Prolonged Corrected QT Interval Is Associated with Lower Incidence of Maternal Hypotension During Spinal Anesthesia in Cesarean Delivery: A Prospective Observational Study
Source: Medicina (Kaunas). 2025 Oct 27;61(11):1925. doi: 10.3390/medicina61111925 (PMC12654321; doi:10.3390/medicina61111925)
Supplement: Supplementary file 1 [file medicina-61-01925-s001.zip › medicina-3918880-supplementary.pdf]

Supplementary Table S1. Patient characteristics of external validation set.

	QTc < 440 ms (n = 44)	QTc ≥ 440 ms (n = 36)	*p-Va*lue
Age (years)	36 (3.3)	34 (4.3)	0.014
Weight (kg)	70.8 (9.1)	70.4 (10.2)	0.858
Height (cm)	161.8 (5.0)	161.1 (4.2)	0.508
Gestational age (weeks)	38.5 (0.7)	38.2 (1.7)	0.244
Potassium (mmol/L)	4.2 (0.2)	4.3 (0.3)	0.048
Calcium (mg/dL)	9.2 (0.4)	9.3 (0.5)	0.296
Apgar score at 1 min	8 (8–9)	8 (8–9)	0.132
Apgar score at 5 min	9 (9–9)	9 (9–9)	0.128

Data are presented as mean (SD), or number (percentage). QTc, corrected QT interval; ms, milliseconds; CSEA, combined spinal-epidural anesthesia; SD, standard deviation; IQR, interquartile range.
